# Circulating α-Klotho Is Elevated in Breast Cancer and Positively Correlates with IGF-1

**DOI:** 10.3390/ijms27209257

**Published:** 2026-10-18

**Authors:** Shira Ben Neriah, Marana Abboud, Alla Berlin, Rina Hemi, Rona Aks, Shiran Shapira, Ido Wolf, Tami Rubinek

**Affiliations:** 1 Department of Oncology, Tel Aviv Sourasky Medical Center, Tel Aviv 6423906, Israel; bnshira1@gmail.com (S.B.N.); maranaabboud@mail.tau.ac.il (M.A.); allan30571@gmail.com (A.B.); drrona64@gmail.com (R.A.); shiranshapira@gmail.com (S.S.); 2 Faculty of Medical and Health Sciences, Tel Aviv University, Tel Aviv 6997801, Israel; 3 Endocrinology Institute, Chaim Sheba Medical Center, Tel Hashomer, Ramat Gan 5262000, Israel; rina.hemi@sheba.health.gov.il

**Keywords:** Klotho, breast cancer, IGF-1, biomarker, tumor suppressor, aging

## Abstract

Breast cancer (BC) is the most commonly diagnosed cancer among women worldwide. Klotho is an anti-aging protein implicated in tumor suppression; however, the relationship between circulating Klotho levels and BC remains unclear. We measured serum Klotho by enzyme-linked immunosorbent assay (ELISA) in 372 participants: 166 patients with BC, 24 patients with ductal carcinoma in situ (DCIS), and 182 controls, and examined its relation to clinicopathological characteristics, including estrogen receptor (ER), progesterone receptor (PR), HER2 and lymph node status, body mass index (BMI), and insulin-like growth factor 1 (IGF-1). Serum Klotho levels were significantly higher in invasive BC and DCIS patients compared with controls (*p* = 0.003 and *p* = 0.001, respectively), whereas no significant difference was detected between invasive BC and DCIS. After adjustment for age, Klotho levels remained significantly higher in BC and DCIS patients than in controls. Klotho levels correlated positively with IGF-1 (*p* = 0.0043), but not with BMI, ER, PR, HER2, or lymph node status. These preliminary findings demonstrate an association between elevated circulating Klotho and BC, as well as a positive relationship between Klotho and IGF-1. Further studies using age-matched cohorts and diagnostic-performance analyses are required to determine whether circulating Klotho has clinical utility as a BC biomarker.

## 1. Introduction

Breast cancer (BC) is the most frequently diagnosed malignancy in women, with a lifetime risk of approximately 1 in 8 among women in the United States. Statistics show that in the past decade, the rate increased by 1% per year [1].

Although incidence has continued to rise, advances in early detection and treatment have substantially improved clinical outcomes. BC is a biologically heterogeneous disease, and its classification now relies not only on histologic features but also on molecular biomarkers such as estrogen receptor (ER), progesterone receptor (PR), HER2, and Ki-67, which are central to prognosis and treatment. Additional alterations, including *BRCA1/2*, *PIK3CA*, and *ESR1*, further support therapeutic decisions [2]. Treatment options have also expanded considerably with surgery, radiotherapy, endocrine therapy, and more, which are now used across both invasive and non-invasive BC depending on disease subtype and stage. However, metastasis and resistance to therapy remain a major challenge [2].

Klotho is a transmembrane protein expressed in the kidneys, brain, and endocrine and exocrine glands and can be cleaved and shed into circulation, where it acts as a hormone [3,4]. Its canonical function is to act as an essential co-receptor of FGF23, thereby regulating phosphate and calcium homeostasis [5,6]. Klotho is an aging-related protein; its levels in humans decrease with age [7], and its deficiency is associated with multiple age-related diseases like chronic kidney disease and reduced β-cell mass in the pancreas, leading to diabetes [8]. Accumulating evidence demonstrates Klotho’s function as a tumor suppressor in BC and other tumors, with decreased expression in tumor tissue compared to the normal adjacent tissue [9]. Loss of Klotho expression was also observed early in breast tumorigenesis [9]. Mechanistically, it was shown that Klotho inhibits key signaling pathways associated with cancer progression [10,11], among them the insulin-like growth factor 1 receptor (IGF-1R) pathway [9,12].

Membrane-bound Klotho can be cleaved by the metalloproteases ADAM10 and ADAM17, releasing soluble Klotho into the circulation. IGF-1 has been shown to promote Klotho shedding [11], suggesting a link between the growth hormone–IGF-1 axis and circulating Klotho levels. Consistent with this relationship, serum Klotho levels are elevated in patients with acromegaly [13] and have been reported to be lower in individuals with growth hormone deficiency [14]. Circulating Klotho has also been reported to decline with age and to be reduced already at early stages of chronic kidney disease [15,16]. Studies examining serum Klotho in patients with cancer, however, have yielded inconsistent findings [17,18,19,20,21,22].

Given the reported anti-aging and tumor-suppressive functions of Klotho, we hypothesized that circulating Klotho levels would differ between patients with BC and healthy controls and might be associated with clinicopathological characteristics and IGF-1 levels. We found higher serum Klotho levels in patients with BC and a modest positive correlation between Klotho and IGF-1. These findings point to changes in circulating Klotho in BC, although their biological basis and clinical significance remain to be determined.

## 2. Results

**Figure 1:**
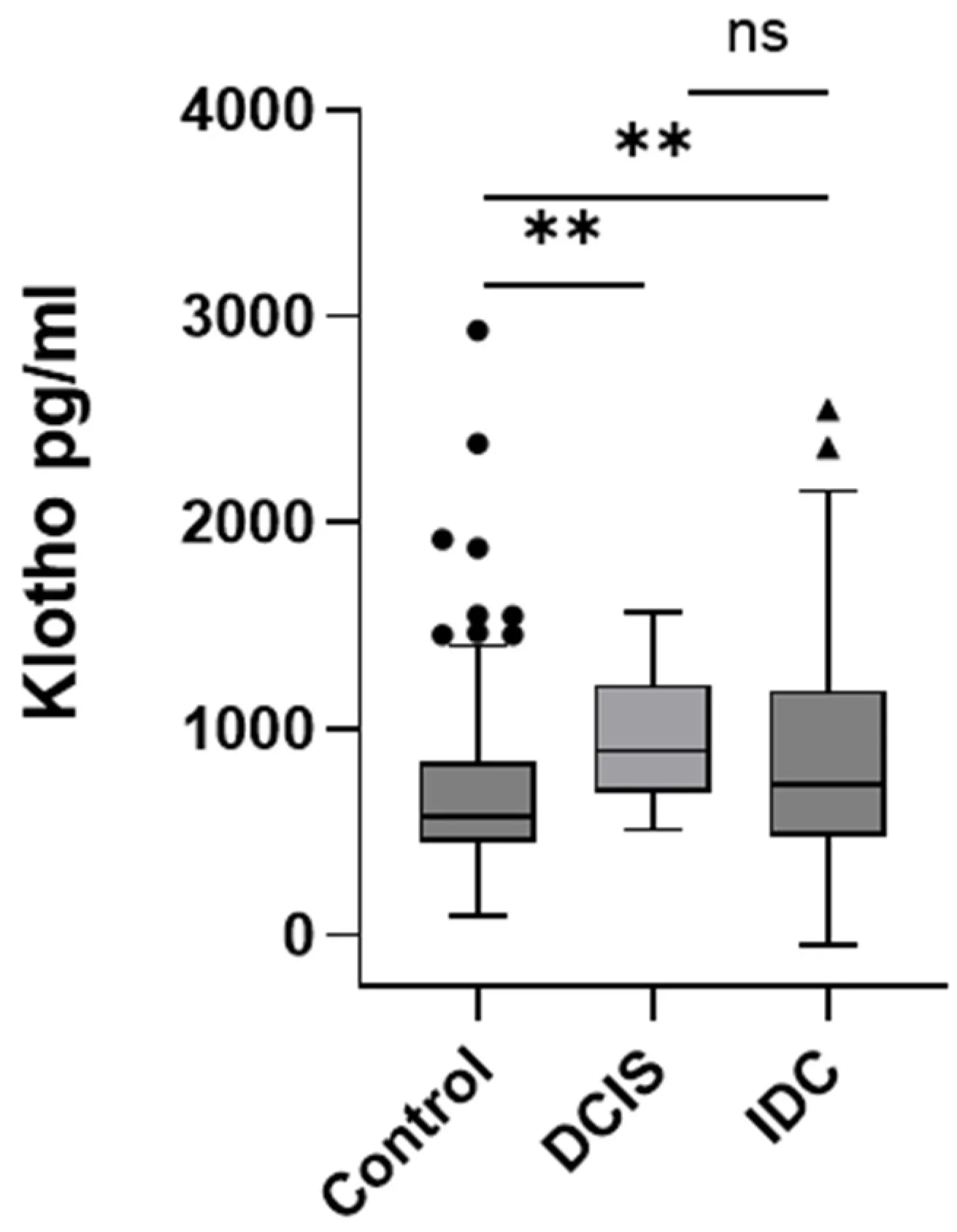
Klotho serum levels across the study groups. Box plots display the serum Klotho levels in control, DCIS, and invasive ductal carcinoma (IDC). Statistical significance was evaluated by the independent-samples Kruskal–Wallis test; ** *p* < 0.01; ns, not significant.

The study included 182 controls, 166 patients with BC, and 24 patients with ductal carcinoma in situ (DCIS). Mean age differed among the three groups (overall *p* < 0.001; Table 1). Median serum Klotho levels were 569.5 pg/mL in controls, 728.9 pg/mL in BC, and 890.8 pg/mL in DCIS (overall *p* < 0.001; Figure 1). Klotho levels were higher in both BC (*p* = 0.003) and DCIS (*p* = 0.001) than in controls, whereas the difference between BC and DCIS was not statistically significant (*p* = 0.151).

**Figure 2:**
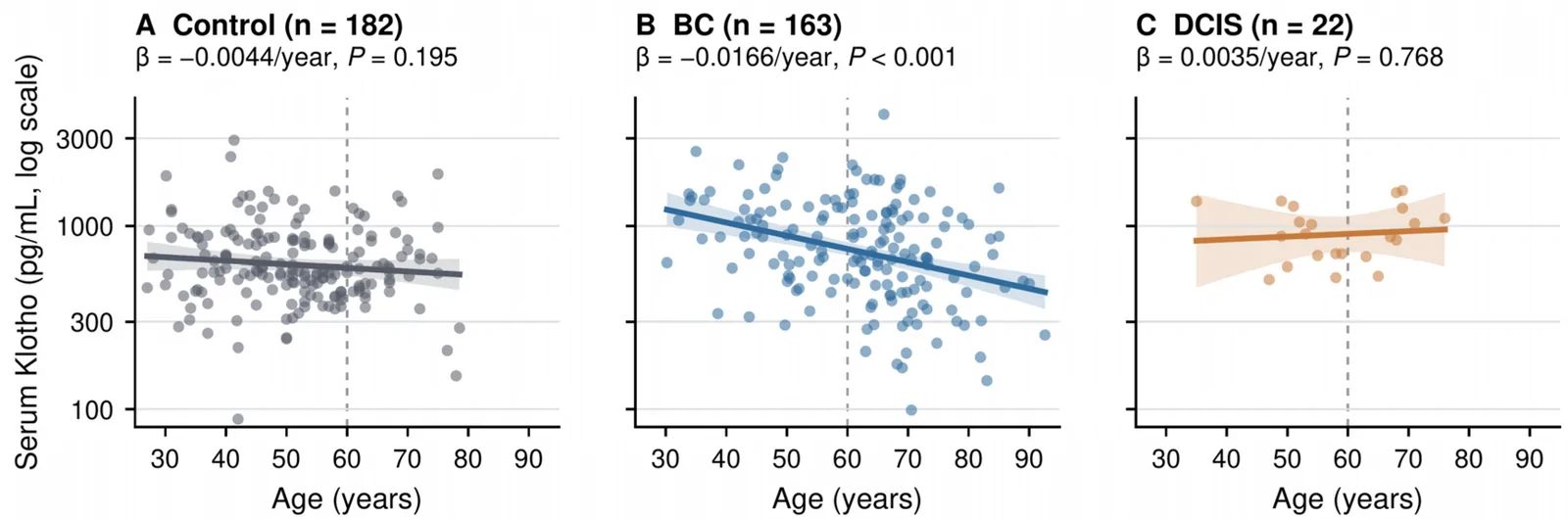
Association between age and serum Klotho levels according to study group. Serum Klotho (pg/mL, log scale) as a function of age in controls (**A**), patients with BC (**B**), and patients with DCIS (**C**). Each point represents one participant. Solid lines and shaded areas show predicted values and 95% confidence intervals from a single multivariable linear regression model of ln(Klotho + 0.01) including study group, age centered at 60 years, and age-by-group interaction terms (*n* = 367). The dashed vertical line marks the reference age of 60 years. Age slopes (β per year) and *p* values are shown for each group. The overall age-by-group interaction was significant (*p* = 0.017).

Given the significant age difference between the groups, we next performed a multivariable linear regression analysis including all participants with valid Klotho and age measurements (*n* = 367). At the reference age of 60 years, adjusted serum Klotho levels were 26.7% higher in patients with BC than in controls (ratio of adjusted geometric means 1.267, 95% CI 1.115–1.440, *p* = 0.0003) and 53.2% higher in patients with DCIS (ratio 1.532, 95% CI 1.195–1.963, *p* = 0.0008), whereas the difference between DCIS and BC was not significant (ratio 1.209, 95% CI 0.948–1.541, *p* = 0.126). The association between age and Klotho differed among the groups (overall age-by-group interaction *p* = 0.017). Age was not significantly associated with Klotho levels among controls (β = −0.0044 per year, *p* = 0.195), whereas older age was associated with lower Klotho levels among patients with BC (β = −0.0166 per year, *p* < 0.001), corresponding to approximately 1.6% per year. No significant age-related association was detected in the smaller DCIS group (β = 0.0035 per year, *p* = 0.768). The age-by-BC interaction was significant (β = −0.0122, *p* = 0.0093), whereas the age-by-DCIS interaction was not (*p* = 0.520; Figure 2).

We next examined whether circulating Klotho levels were associated with tumor receptor status. Among patients with available receptor data, 116/143 (81.1%) had ER-positive tumors, 83/143 (58.0%) had PR-positive tumors, and 19/139 (13.7%) had HER2-positive tumors. Median circulating Klotho levels were numerically lower in patients with ER-positive than ER-negative tumors (689.1 vs. 819.9 pg/mL), PR-positive than PR-negative tumors (677.8 vs. 716.1 pg/mL), and HER2-positive than HER2-negative tumors (666.9 vs. 717.3 pg/mL). However, none of these differences were statistically significant (ER, *p* = 0.80; PR, *p* = 0.85; HER2, *p* = 0.79; Table 2). Among invasive BC patients with available lymph node data (*n* = 81), Klotho levels were numerically higher in lymph node-positive cases (median 648.9 pg/mL) compared with lymph node-negative cases (median 567.3 pg/mL), although this difference did not reach statistical significance (*p* = 0.54; Table 3).

**Figure 3:**
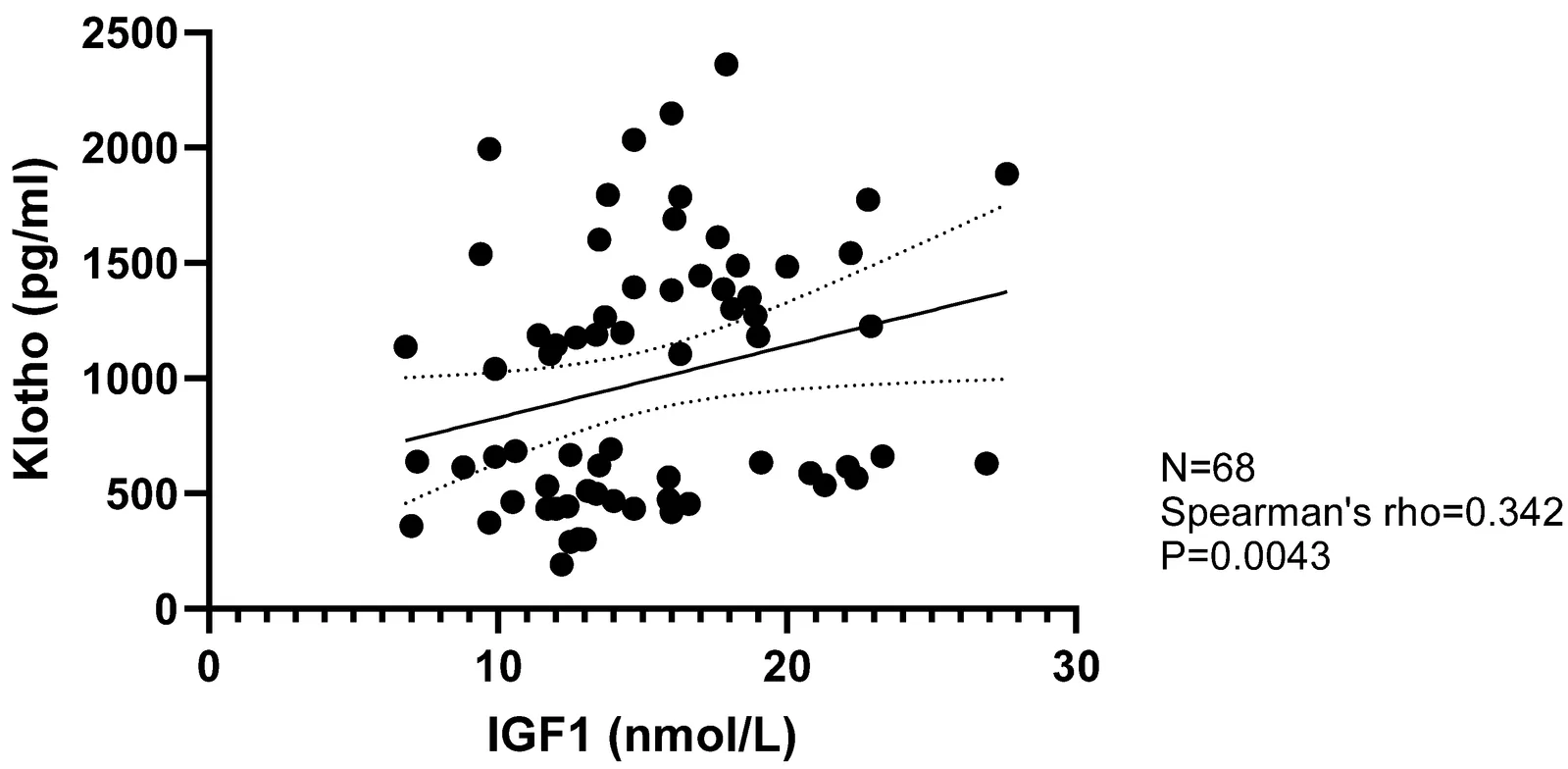
Association between circulating α-Klotho and serum IGF-1 levels in breast cancer patients. Each point represents an individual participant. The solid line indicates the fitted linear trend, and the dotted lines represent the 95% confidence interval. Correlation was evaluated using Spearman’s rank correlation coefficient (*r*_s_ = 0.342, *p* = 0.0043, *n* = 68).

**Figure 4:**
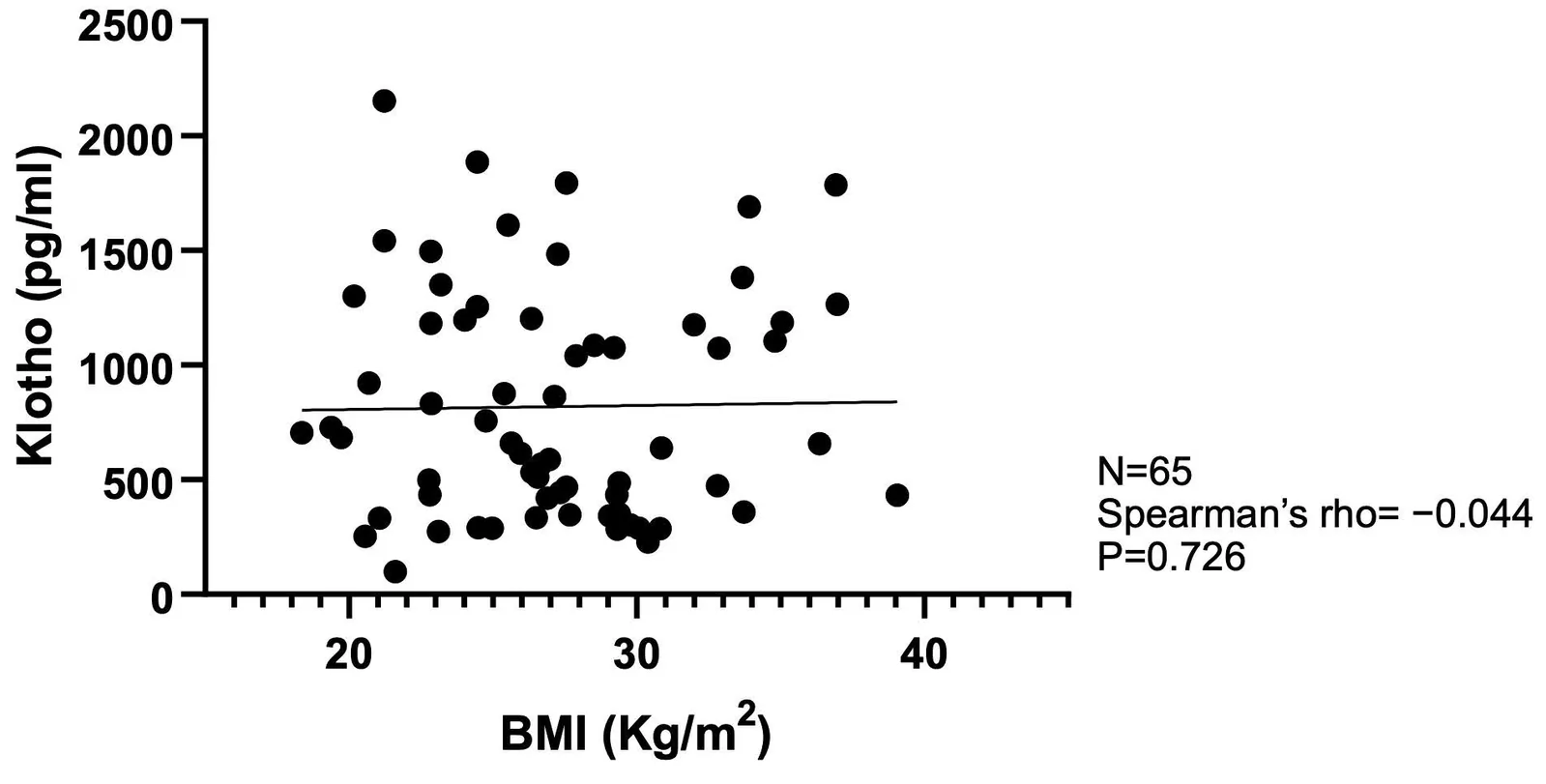
Correlation between Klotho serum levels and BMI. Scatter plot showing serum α-Klotho concentrations versus BMI (kg/m^2^) (*n* = 65). Each point represents an individual participant. The solid line indicates the fitted linear trend. Spearman’s correlation analysis revealed no significant correlation between serum α-Klotho levels and BMI (ρ = −0.044, *p* = 0.726).

IGF-1 is the main known regulator of Klotho in the circulation [9,11]; therefore, its serum levels were evaluated in BC patients. Interestingly, we found a positive and significant correlation between IGF-1 and Klotho levels, with a correlation coefficient of 0.342 (*p* = 0.0043; Table 4, Figure 3). Among the 68 patients with evaluable IGF-1 measurements, age was inversely correlated with serum IGF-1 levels (Spearman’s *r*_s_ = −0.395, *p* < 0.001). BMI measured in 65 BC patients was not significantly associated with Klotho serum levels (Table 4, Figure 4).

## 3. Discussion

In this study, we evaluated the association between serum Klotho levels and BC in a relatively large cohort of patients. We observed elevated serum Klotho levels in patients with invasive BC and DCIS compared with controls, whereas Klotho levels were similar between invasive BC and DCIS. Notably, the patients with BC were older than the controls. Because circulating Klotho levels have generally been reported to decline with age [23], this age difference would be expected to bias the comparison toward lower, rather than higher, Klotho levels in the cancer group. Thus, the observed elevation in circulating Klotho is unlikely to be explained by the age imbalance between the groups and may, if anything, have been attenuated by it. Nevertheless, the significant age-by-group interaction indicates that the association between age and Klotho differs according to disease status: older age was associated with lower Klotho levels among patients with BC, but not among controls or patients with DCIS, and these findings should be interpreted accordingly.

Our finding of increased serum Klotho levels in patients with breast cancer is consistent with several previous studies. Most notably, a recent study by Renu et al. included 263 newly diagnosed, treatment-naïve patients with breast cancer and 100 healthy controls and reported significantly higher circulating Klotho levels in the cancer group (*p* < 0.0001) [17]. Using surface plasmon resonance, the authors also detected elevated serum ADAM10, a metalloproteinase involved in Klotho shedding, and observed a positive correlation between Klotho and ADAM10 levels (r = 0.449, *p* < 0.0001), supporting enhanced Klotho shedding as a possible contributor to elevated circulating Klotho. In addition, Klotho levels were higher in patients with metastatic disease, larger tumors, advanced-stage disease, and ER-positive tumors, whereas no significant associations were observed with lymph node, PR, or HER2 status. In our cohort, we similarly found no association with lymph node, PR, or HER2 status, but, unlike Renu et al., we did not detect a significant association with ER status. These differences may reflect variation in cohort size and composition, disease stage, age, and measurement methodology. In a longitudinal subset of 100 patients, Renu et al. further showed that serum Klotho levels decreased after three months of treatment and were lower in patients who achieved a complete or partial response than in those with stable or progressive disease. Their ROC analysis also showed discrimination between patients with breast cancer and healthy controls (AUC = 0.938), although these diagnostic findings require external validation. Direct comparison of absolute Klotho concentrations between that study and ours is limited because Renu et al. used surface plasmon resonance, whereas we used ELISA. Nevertheless, both studies independently demonstrate elevated circulating Klotho in patients with breast cancer.

Elevated serum Klotho levels were detected in other malignancies. A study including 20 hepatocellular carcinoma patients and 29 healthy controls reported higher Klotho levels in cancer patients compared with controls (mean = 291.6 vs. 186.06 pg/mL, respectively) [18]. Similarly, a comparison between 40 patients with esophageal cancer and 40 healthy controls demonstrated elevated Klotho levels in cancer patients (461.5 vs. 239.37 pg/mL, respectively) [19]. Although both studies included a relatively small sample size, they consistently reported significantly higher mean serum Klotho levels in cancer patients. In contrast, a study of newly diagnosed lung cancer patients indicated no significant difference in Klotho levels between cancer patients and controls [20]. Furthermore, a study of diffuse large B cell lymphoma that evaluated serum levels in a small patient cohort indicated lower Klotho levels (628.54 pg/mL) compared with healthy controls (818.87 pg/mL, *p* = 0.045) [21]. Similar findings were observed in renal cell carcinoma, where a study including a larger number of cancer patients (*n* = 160) reported lower serum Klotho levels than in healthy subjects and identified a significant correlation with renal cell carcinoma progression [22]. Taken together, these apparently discordant findings suggest that circulating Klotho is not uniformly altered across malignancies and may be influenced by tumor-specific and host-related factors that regulate Klotho expression and shedding. One potential modifier of circulating Klotho levels is the systemic IGF-1 milieu.

In the present cohort, serum Klotho levels correlated positively with circulating IGF-1. Although the strength of the correlation was modest, this finding is consistent with previous evidence linking the GH/IGF-1 axis to circulating Klotho levels. We previously demonstrated that IGF-1 directly stimulates Klotho shedding through activation of the AKT–mTOR pathway [11], while insulin has also been shown to promote ADAM10- and ADAM17-mediated cleavage and release of Klotho from the cell surface [24]. Moreover, higher circulating IGF-1 levels have been associated with an increased risk of breast cancer, and some studies have reported higher total IGF-1 concentrations in patients with breast cancer than in healthy women [25,26,27]. Thus, differences in circulating IGF-1 levels or IGF-1 pathway activity among cancer types and patient populations may contribute to the apparently inconsistent changes in serum Klotho reported across malignancies. Cohorts characterized by higher circulating IGF-1 may exhibit enhanced Klotho shedding and consequently higher serum Klotho levels, whereas this response may be absent in malignancies not associated with increased systemic IGF-1 activity. Given the inhibitory effects of soluble Klotho on IGF-1R signaling [10,12], increased Klotho shedding could also represent a counter-regulatory response to enhanced IGF-1 activity [11,24]. However, the cross-sectional association observed in our study cannot establish the direction or causality of this relationship, and this proposed mechanism requires further investigation.

Another important aspect of studying circulating Klotho levels in patients with cancer is their potential relevance to the development of Klotho-based anticancer therapies. The observation that circulating Klotho levels are elevated in patients with BC does not necessarily contradict the potential use of Klotho as a therapeutic agent. Endogenous serum concentrations may reflect a compensatory host response or increased shedding associated with the disease, and immunoassay-based measurements do not establish the molecular form, biological activity, stability, or intratumoral availability of circulating Klotho. In contrast, experimental studies have shown that Klotho overexpression or treatment with exogenous soluble Klotho or its active KL1 domain inhibits breast and pancreatic cancer cell growth in vitro and suppresses tumor growth in vivo, at least partly through inhibition of IGF-1R signaling and modulation of FGF signaling [9,12]. In our previous pancreatic cancer study, delivery of an AAV vector encoding the secreted Klotho isoform inhibited established xenografts, whereas systemic treatment with recombinant soluble Klotho prolonged survival in a genetically engineered KPC model [28]. These findings suggest that therapeutic activity may require sustained pharmacological exposure exceeding the biologically active Klotho available within the tumor microenvironment, even when total circulating Klotho is elevated. Future studies should therefore define the relationship between measured serum Klotho, biologically active Klotho, and intratumoral Klotho exposure and identify the patients most likely to benefit from treatment. Potential selection criteria may include low tumor Klotho expression, activation of IGF-1R- or FGF-dependent signaling, or evidence of insufficient functional Klotho activity despite elevated circulating levels. Tumor-targeted or local delivery approaches could also be explored to increase intratumoral exposure while limiting systemic effects. However, the present observational findings do not establish therapeutic efficacy or define a therapeutic concentration, and these possibilities require dedicated preclinical pharmacokinetic and pharmacodynamic studies.

Several limitations should be acknowledged. The control group was not age-matched to the cancer groups, although age was addressed in the multivariable analysis. Clinicopathological information obtained from hospital records was incomplete, resulting in smaller sample sizes for analyses of receptor status, lymph node involvement, BMI, and IGF-1. Information on disease stage, renal function, metabolic status, concurrent medications, and treatment exposure was unavailable or incomplete, and these factors may influence circulating Klotho levels. Sociodemographic and lifestyle data were not collected, and residual confounding by these or other unmeasured factors cannot be excluded. The relatively small DCIS cohort and several small breast cancer subgroups reduced the power to detect clinically relevant associations. In addition, the single-center, cross-sectional design precludes conclusions regarding causality, temporal changes, prognosis, or disease progression. Finally, diagnostic performance was not evaluated; therefore, the potential clinical utility of circulating Klotho as a breast cancer biomarker requires validation in independent, age-matched cohorts using appropriate diagnostic-performance analyses.

## 4. Materials and Methods

### 4.1. Study Population

This was a preliminary, single-center, hospital-based cross-sectional study using banked serum samples. The study population consisted of a study cohort and a control group. All personal identifiers from the study population were de-identified to ensure patient privacy and confidentiality. The study cohort comprised 190 women diagnosed with BC (*n* = 166) and DCIS (*n* = 24), histologically confirmed by the institution’s pathology department. All serum samples from this cohort were obtained from the tissue bank of our tertiary medical center and were collected prior to the initiation of any oncological intervention, including chemotherapy, radiation, or hormonal therapy.

The control group included 182 healthy women with no history of cancer. The median age of this group was 52 years (range: 27–78.6 years). The controls were not matched to the patient groups by age; therefore, age was accounted for in the multivariable regression analysis. These serum samples were sourced from within the same institution: 12 samples were provided by the institutional tissue bank, while the remaining 170 samples were randomly selected from the serum collection of the Health Promotion and Integrated Cancer Prevention Center (ICPC) at the Tel Aviv Medical Center.

The study was approved by the Institutional Human Investigations (Helsinki) Committee, and all participants provided written informed consent for the use of their biological materials and corresponding medical data in research. Following this protocol, clinical and pathological data, including BMI, histological diagnosis, ER/PR status, HER2 status, and lymph node involvement, were retrieved from institutional records. Clinicopathological data were not available for all patients; therefore, analyses of individual clinical and pathological variables were restricted to patients with available data, and the corresponding sample size is reported for each analysis.

### 4.2. Klotho Serum Measurement

Blood samples were collected following an overnight fast and centrifuged at 2700 rpm for 15 min. Serum was separated and stored at −80 °C until analysis. Serum α-Klotho concentrations were measured using an ELISA kit (Immuno-Biological Laboratories Co., Fujioka, Japan) according to the manufacturer’s instructions [29]. One sample from the BC group was excluded because of a technical assay error that produced an invalid negative concentration. Consequently, Klotho measurements from 165 of the 166 patients with BC were included in the analysis.

### 4.3. IGF-1 Serum Measurement

Serum IGF-1 was measured in 69 BC samples for which sufficient serum remained. One measurement was excluded because of a pipetting error during the assay, leaving 68 valid IGF-1 results for analysis. IGF-1 concentrations were quantified at the Endocrinology lab at the Institute of the Chaim Sheba Medical Center. Serum IGF-1 concentrations were determined by a solid-phase enzyme-linked chemiluminescent immunoassay (Immulite 2000 system, Siemens Healthcare Diagnostics, Inc., Tarrytown, NY, USA). The lower limit of detection (LoD) of the assay was 2 nmol/L. The intra- and inter-assay variation coefficients were 3.3–5.5% and 4.1–7.3%, respectively.

### 4.4. Statistical Analysis

Statistical analyses were performed using SPSS (version 29; IBM Corp., Armonk, NY, USA), R (version 4.4.0; R Foundation for Statistical Computing, Vienna, Austria), and Python (version 3.12.14; Python Software Foundation, Wilmington, DE, USA; NumPy version 2.3.5, pandas version 2.2.3, SciPy version 1.17.0, and Matplotlib version 3.10.8). Data distribution was assessed using the Shapiro–Wilk test and visual inspection of histograms.

Differences in Klotho levels across the three study categories (healthy controls, DCIS, and BC) were analyzed using the independent-samples Kruskal–Wallis test. In cases where the overall test was significant, pairwise comparisons were performed using the Mann–Whitney U test, with Bonferroni correction applied for multiple comparisons. The same approach (Mann–Whitney U test) was used to compare Klotho levels between the ER-positive and ER-negative, PR-positive and PR-negative, HER2-positive and HER2-negative, and LN-positive and LN-negative groups.

Age was compared among the control, BC, and DCIS groups using Welch’s one-way analysis of variance (ANOVA).

The relationship between Klotho levels and continuous variables, including BMI and IGF-1, was evaluated using Spearman’s rank correlation coefficient (rho). To evaluate age-adjusted differences in serum Klotho levels among the study groups, a multivariable linear regression model was fitted with log-transformed Klotho [ln(Klotho + 0.01)] as the dependent variable. Study group (controls as the reference category, BC, and DCIS), age centered at 60 years, and age-by-group interaction terms were included as independent variables, and the overall age-by-group interaction was evaluated using a joint F-test. The model included 367 participants with valid Klotho and age measurements (182 controls, 163 patients with BC, and 22 patients with DCIS). Group coefficients were exponentiated and are presented as ratios of adjusted geometric means with 95% confidence intervals (CIs). The association between age and serum IGF-1 levels was evaluated using Spearman’s rank correlation coefficient.

Continuous variables are presented as mean ± standard deviation (SD) or median [interquartile range, Q1–Q3], as appropriate. All statistical tests were two-sided, and a *p*-value < 0.05 was considered statistically significant.

## 5. Conclusions

Circulating Klotho levels were elevated in patients with invasive BC and DCIS, although the magnitude of these differences varied with age. Klotho also correlated positively with IGF-1, supporting a potential link between the IGF-1 axis and Klotho shedding. Together with recent independent findings, our results strengthen the association between elevated circulating Klotho and BC. Further age-matched, longitudinal studies combining serum and tumor measurements are required to clarify its biological significance and evaluate its potential diagnostic and therapeutic relevance.

## Figures and Tables

**Table 1:** Age and serum Klotho levels by study group.

	Control (*n* = 182)	BC (*n* = 166) ^3^	DCIS (*n* = 24)	*p*
Age (years), mean ± SD	51.5 ± 11.8	61.8 ± 13.2	58.5 ± 10.0	<0.001
Serum Klotho (pg/mL), median [Q1–Q3]	569.5 [448.9–839.1]	728.9 [481.7–1184.2] ^1^	890.8 [692.2–1213.7] ^2^	<0.001

^1^ BC versus controls: *p* = 0.003; ^2^ DCIS versus controls: *p* = 0.001. These *p* values were adjusted for multiple comparisons. For BC versus DCIS, the adjusted *p* value was 0.151. In the Klotho row, *p* < 0.001 refers to the overall comparison among the three groups. ^3^ Klotho measurements were available for 165 of 166 patients in the BC group; one negative value was excluded.

**Table 2:** Receptor status in breast cancer patients and its association with circulating Klotho levels.

Receptor	Status	N (%)	Klotho Median (pg/mL)	*p *-Value
ER (*n* = 143)	ER+	116 (81.1%)	689.1	0.80
	ER−	27 (18.9%)	819.9	
PR (*n* = 143)	PR+	83 (58%)	677.8	0.85
	PR−	60 (42%)	716.1	
HER2 (*n* = 139)	HER2+	19 (13.7%)	666.9	0.79
	HER2−	120 (86.3%)	717.3	

**Table 3:** Lymph node status and its association with circulating Klotho levels.

Lymph Node Invasion (*n* = 81)	N (%)	Klotho Median (pg/mL)	*p *-Value
LN+	20 (24.7%)	648.9	0.54
LN−	61 (75.3%)	567.3	

**Table 4:** IGF-1 and BMI measurements and their correlation with circulating Klotho levels.

	IGF-1 (nmol/L)	BMI (kg/m^2^)
N	68	65
Mean ± SD	15.17 ± 4.58	27.1 ± 4.8
Spearman’s rho	0.342	−0.044
*p*-value	0.0043	0.726

## Data Availability

The data presented in this study are available upon request from the corresponding author. The data are not publicly available due to privacy and ethical restrictions.
